# Implementing a knowledge application program for anxiety and depression in community-based primary mental health care: a multiple case study research protocol

**DOI:** 10.1186/1748-5908-8-26

**Published:** 2013-03-04

**Authors:** Pasquale Roberge, Louise Fournier, Hélène Brouillet, Catherine Hudon, Janie Houle, Martin D Provencher, Jean-Frédéric Lévesque

**Affiliations:** 1 Département de médecine de famille et de médecine d’urgence, Faculté de médecine et des sciences de la santé, Université de Sherbrooke, 3001, 12e Avenue Nord, Sherbrooke, Québec, Canada; 2 Institut national de santé publique du Québec, 190, boul. Crémazie Est, Montréal, Québec, Canada; 3 Centre de recherche du Centre hospitalier universitaire de Montréal (CRCHUM), Pavillon Édouard-Asselin, 264, boul. René-Lévesque Est, Montréal, Québec, Canada; 4 Département de psychologie, Université du Québec à Montréal, Pavillon J.A. De Sève, 305 rue Christin, Montréal, Québec, Canada; 5 École de psychologie, Université Laval, Pavillon Félix-Antoine-Savard, 2325, rue des Bibliothèques, Québec, Québec, Canada

**Keywords:** Depression, Anxiety disorders, Primary care, Quality of care, Quality improvement, Mental health care, Quality indicators, Knowledge application

## Abstract

**Background:**

Anxiety and depressive disorders are increasingly recognized as a health care policy priority. Reducing the treatment gap for common mental disorders requires strengthening the quality of primary mental health care. We developed a knowledge application program designed to improve the organization and delivery of care for anxiety and depression in community-based primary mental health care teams in Quebec, Canada. The principal objectives of the study are: to implement and evaluate this evidence-based knowledge application program; to examine the contextual factors associated with the selection of local quality improvement strategies; to explore barriers and facilitators associated with the implementation of local quality improvement plans; and to study the implementation of local quality monitoring strategies.

**Methods:**

The research design is a mixed-methods prospective multiple case study. The main analysis unit (cases) is composed of the six multidisciplinary community-based primary mental health care teams, and each of the cases has identified at least one primary care medical clinic interested in collaborating with the implementation project. The training modules of the program are based on the Chronic Care Model, and the implementation strategies were developed according to the Promoting Action on Research Implementation in Health Services conceptual framework.

**Discussion:**

The implementation of an evidence-based knowledge application program for anxiety and depression in primary care aims to improve the organization and delivery of mental health services. The uptake of evidence to improve the quality of care for common mental disorders in primary care is a complex process that requires careful consideration of the context in which innovations are introduced. The project will provide a close examination of the interplay between evidence, context and facilitation, and contribute to the understanding of factors associated with the process of implementation of interventions in routine care. The implementation of the knowledge application program with a population health perspective is consistent with the priorities set forth in the current mental health care reform in Quebec. Strengthening primary mental health care will lead to a more efficient health care system.

## Background

Anxiety and depressive disorders are increasingly recognized as a health care policy priority as they are the most common mental disorders among the general population and in primary care [1-5]. Lifetime prevalence is approximately 6.7% for major depression [4] and 16.6% for anxiety disorders [3]. These disorders are associated with significant psychological distress, and functional and social impairment [5,6]. People living with these disorders present a high risk of comorbidity, as anxiety and depressive disorders are frequently occurring with other mental disorders as well as chronic physical illness [7-10]. This co-occurrence is also proportional to the severity of disability, persistence of symptoms and deterioration of individuals’ health [7-9]. Efficient management of anxiety and depressive disorders could lead to a reduction in the social and economic costs of mental disorders [11-14]. Global health policies are increasingly concerned with improving public mental health [15,16], and the role of primary care in the recognition and treatment of common mental disorders has significantly evolved since the 1990s [17,18]. While the integration of mental health into primary care is paramount to improving access to mental health care, the current reforms require support to strengthen the quality of primary mental health services across a wide variety of contexts [17].

Research on the treatment gap for common mental disorders has shown that clinical practices do not keep pace with the ever-growing knowledge regarding optimal anxiety and depression management. This gap is a key issue in mental health services research, and concerted effort is required to further knowledge on the implementation of evidence-based practices [19]. Although pharmacological and psychological treatments for anxiety and depression have existed for several years now [20-22], it has been established that only a minority of anxiety or depression sufferers are diagnosed and treated according to clinical practice guidelines’ recommendations [23-28]. Access and equity issues have also been raised, in which individual factors such as age, education and mental health insurance coverage have been associated with access to care and treatment adequacy [23,28-32]. Several individual, social, professional and systemic factors contribute to this situation, such as low help-seeking and utilization of mental health services for common mental health problems, under-detection of anxiety and depressive disorders in primary care, limited access to evidence-based treatments, particularly psychotherapy, and lack of treatment intensification when required [1,33-35].

Research has evaluated several interventions to improve the quality of primary mental health care for common mental disorders, predominantly for depression. The broad spectrum of strategies used to implement change in clinical practice includes simple and inexpensive professional interventions, financial interventions, and organizational interventions centred on patients, care providers, and the health care system [36]. It is well recognized that isolated educational or organizational strategies, such as passive dissemination of clinical practice guidelines or systematic screening, only minimally impact patient outcomes [37,38]. Furthermore, consultation-liaison models do not appear sufficient as stand-alone interventions to improve patient outcomes at the population level [39]. Numerous studies show that implementing complex multimodal intervention strategies that include both organizational and educational activities can result in improvements to primary care quality and patient health [37,38,40-42]. Among complex models of quality improvement, collaborative care has been shown to be more effective than usual care in numerous trials for depression, and in some trials for anxiety disorders [38,40,42,43]. Models of collaborative care typically include active collaboration between primary care providers and mental health specialists, predominantly psychiatrists, and the role of a case manager [41,44,45]. The key functions of the case manager may include patient education, self-management support, systematic follow-up, clinical management, low intensity interventions, and care coordination [41,46]. Principles of stepped care have also been introduced in recent years to improve efficiency by modulating services according to patients’ characteristics. Stepped care involves continuous and systematic assessment of patient outcomes, as well as collaboration between primary care and specialised mental health service [35,47-49]. Current knowledge of the most promising active ingredients in these complex strategies is mature enough to implement them into real world clinical practice settings.

There is growing interest in introducing the notion of long-term management in the treatment of depression [35]. The Chronic Care Model (CCM) is the leading model for the management of long-term chronic conditions, a framework for improvement with a population-based approach that aims to provide planned, proactive, patient-centred, and evidence-based care delivery [50,51]. It involves changes at the system, community, organization, professional and patient level. A growing number of evaluative studies indicate that quality improvement programs based on the CCM lead to improvements in both health care processes and patient health [52,53]. The CCM offers a framework for change that is compatible with complex interventions for common mental disorders. Thus, the model seems particularly useful to structure anxiety and depression care organization, as these are often relapsing and tend to become chronic.

### Primary mental health care services in the province of Quebec, Canada

Primary care services have become the key pillar of the mental health care system in Quebec. As of 2005, Quebec’s ministerial policies for mental health have focused on improving the primary care management of mental disorders and achieving an optimal hierarchy of care [54,55]. The mental health strategy included the implementation of community-based primary mental health care teams (CMHTs) throughout the province in each of the 94 Health and Social Services Centres, established within local services networks in each region, with a population responsibility and the management of a gateway for mental health services to ensure access and continuity of care. The team typically comprises a health care administrator, clinical coordinators, psychologists, social workers, nurses, psycho-educators and, occasionally, general practitioners. It is expected as part of the reform that these CMHTs work in collaboration with their local services networks, including primary care clinics, as well as other health care professionals (*e.g.*, psychologists in private practice, community resources, hospital’s emergency departments), to meet the mental health care needs of the population. From a population health perspective, we postulate that patients with common mental disorders should be a priority in the CMHTs’ organisation and delivery of care. Consequently, they need to be supported to improve the quality of care for anxiety and depression, to be informed about the best available research evidence, and to use the finite resources of the public health care system in the most effective and efficient manner.

Research on the implementation of evidence-based practice has typically been conducted in controlled conditions, in medical clinics. Considering that Quebec’s primary CMHTs are not established on a medical practice foundation, and considering the relative lack of knowledge on barriers and facilitators to the implementation of change in that innovative context, we developed a knowledge application program specifically designed to support CMHTs in the organization and delivery of care for anxiety and depression. We grounded the knowledge application program on the CCM to ensure that the implementation of change in clinical practice would be supported by rigorous evidence-based data at the patient, clinical and organisational levels. We also relied on the Promoting Action on Research Implementation in Health Services (PARiHS) conceptual framework developed by Kitson and her collaborators [56,57] to design our implementation strategies. According to this model, successful implementation stems from the dynamic and simultaneous relationship between three elements: evidence, context and facilitation [56]. The dimension of ‘evidence’ refers to scientific robustness, as well as clinical experience and patient preferences. The dimension of ‘context’ takes into account the role of the context, culture, leadership and evaluation in the implementation of interventions in clinical practice**.** ‘Facilitation’ refers to the process of supporting and enabling the implementation through internal and external facilitators. Concerted efforts are essential to facilitate the uptake of evidence-based health care interventions, and research must focus on the complex process of implementing health service organization innovations and ensuring their sustainability [58]. This pragmatic and participative approach aims to study the extent to which clinical and organisational interventions assessed in controlled conditions can be implemented in multiple clinical practice contexts in Quebec. Studies that offer a system assessment perspective on implementation are valuable to improve our comprehension of factors that foster or hinder the implementation of interventions in real world practice in primary care [59,60].

### Objectives

1. To implement and evaluate a knowledge application program for anxiety and depression in six community-based primary mental health care settings;

2. To examine the contextual factors associated with the selection of quality improvement strategies integrated in the local quality improvement plans;

3. To study barriers and facilitators associated with the level of implementation of the local quality improvement plans;

4. To assess the impact of the clinical information systems component of the knowledge application program on the development and implementation of local quality monitoring strategies.

## Methods

### Study design

The research design is a mixed-methods prospective multiple case study [59,61]. A case study design has been selected to thoroughly examine the implementation of the knowledge application program within each CMHT and associated primary care clinic, and to examine the complex interplay between context, evidence and facilitation. Numerous variables determine the successful implementation of organizational and clinical changes in mental health care, and the multiple cases allow for the replication of results among diverse contexts. Case study research could complement current quality improvement research by contributing to understanding of contextual factors associated with the success or failure of quality improvement projects [62].

### Ethical approval

The Research Ethics Committee of the Montréal Health and Social Services Centres Agency, acting as the primary ethics committee for the multicentre project, has approved this study. The six local ethics authorities have endorsed the decision. All local committee members will be asked to sign a written consent form. The confidentiality of participating patients will be protected with coded and anonymous processing of data.

### Setting

#### Community-based mental health care teams

The six CMHTs were selected from the 94 Health and Social Services Centres in the province of Québec, Canada, according to diversity in terms of their local health network’s size, resources, and geographic environment (urban, semi-urban, or rural). A purposeful sampling strategy was selected to examine the implementation process in a variety of contexts. The inclusion of a CMHT in the sample was conditional to: the identification of a primary care medical clinic, usually a family medicine group [63], that would agree to collaborate to the research project; and the commitment to collect patient data as part of the implementation of the quality improvement program. The main analysis unit (case) is composed of the multidisciplinary CMHT. The general characteristics of the six CMHTs included in the sample are presented in Table 1. Two of the CMHTs had participated in a previous quality improvement project conducted from 2008 to 2010. For each of the six CMHTs, organisation and clinical participants to the quality improvement research project included an administrative leader mandated to ensure coordination of the implementation at the local level and an interdisciplinary local working group (see Table 1).

**Table 1:** Organisation profiles and characteristics

	**Organisations**					
**Organisational characteristics**	**1**	**2**	**3**	**4**	**5**	**6**
Population	139, 000	140, 000	220,000	245,000	75,000	55,000
Location						
Urban	X	X				
Semi-urban			X	X	X	
Rural						X
University-affiliated	X		X		X	
Hospital integrated to the CSSS		X	X	X	X	X
**Full-time personnel employed at the CMHT**	30.1	22.3	6.8	18	12.3	5.6
Nurses	5.6	4	3	5.6	2.8	2
Social workers	12.5	9.8	2	4.8	3.4	1.8
Psychologists	10.4	7.2	1.8	6.8	5.1	1.8
Other health professionals	1.6	0.3		0.8	1	
	OT	OC		HRA	ED	
GPs	X					
Consulting psychiatrists	X	X	X	X	X	X
Clinical coordinator	X	X	X		X	
**Description of the associated medical clinic**	1	1	2	1	1	1
Family medicine group	X		X	X	X	X
Family medicine unit	X	X			X	
Network clinic		X				
**Composition of local work committee (total)**	7	10	14	10	11	11
Health care administrator	1^*^	1^*^	1	1^*^	4^*^	1
GPs	1	1	2	2	1	1
Nurses	1	2	2	2	2	3
Psychologists	2	2	2	2	1	2
Social workers		2	2	1	1	2
Clinical coordinator	1		1		2	
Psychiatrists			2	2		
Project manager/Research agent			2^*^			1^*^
Other health professionals						1
Members of other organisations	1	2			1	

**Abbreviations:** CSSS: *Centre de santé et de services sociaux*/Health and Social Services Centre; CMHT: Community-based mental health care team; OT: Occupational therapist; CO: Community organizer; HRA: Human relation agent; ED: Educator.

* Local leader.

#### Description of the intervention

The study builds on a knowledge application program that was developed in a previous project for anxiety and depression in CMHTs [64]. We developed the knowledge application program based on the determinants of a successful implementation of change in clinical practice outlined in the PARiHS framework [56,57]. The program comprises two main phases. The first phase involves the presentation of the knowledge application modules and the development of local quality improvement plans in each local working group, while the second phase consists of the implementation of the quality improvement plans at the local level.

### Phase 1: The knowledge application program

#### The meetings and training modules

The 10 modules of the knowledge transfer phase are presented through six meetings and training sessions with local working groups. The objectives and overview of the modules of the knowledge application phase are presented in Table 2. The knowledge application program is founded on evidence-based data for patient, professional and organizational interventions for common mental disorders. Strategies for improvement related to each of the six components of the CCM are addressed throughout the modules: Health System Organization; Community Resources and Policies; Delivery System Design; Decision Support; Clinical Information Systems; Self-Management Support. As an illustration of the contents related to each component of the model, the ‘Delivery System Design’ component includes educational and discussion material on collaborative care and stepped care, while ‘Decision Support’ presents a number of clinical practice guidelines and decision support tools. Considering that local working groups are required to develop and implement a strategy to collect patient data as part of the ‘Clinical Information Systems’ component of the CCM, an overview of that specific improvement strategy will be presented.

**Table 2:** The knowledge application program

**Module**	Title, chronic care model components [CCM] *, Objective	**Content**
1	**The context[CCM: HSO, CRP]:** To understand the principles that support the implementation of the knowledge application program, the bases of the knowledge application program, and the key policies and orientations that directed the structuring of mental health services in Quebec, Canada.	1. Common mental disorders in primary care
		2. The Chronic Care Model [50,51]
		3. Quality of care for depressive and anxiety disorders
		4. The organization of the health care system
2	**The knowledge application program[CCM: HSO, DSD, DS, CIS, SM, CRP]:** To enable participants to understand the scope of the knowledge application program and know the different stages of its deployment.	1. Project and objectives
		2. Knowledge application program
		3. Developing and implementing a local quality improvement plan
3	**The implementation of changes in clinical practice [CCM: HSO, DSD, DS]:** To provide participants with a systemic view of the implementation of changes in an organizational context. To understand the role played by the local working committee and identify key stakeholders.	1. The conceptual framework: the PARiHS model [56,57]
		2. Knowledge application in the Health and Social Services Centre context
		3. The Knowledge-to-action cycle [65]
4	**The stepped-care model [CCM: DSD, CIS]:** Participants will be guided by the principles and concepts presented in order to review their services and care pathways; the role and responsibilities of the various care providers throughout the care pathway will also be reviewed.	1. Delivery system design
		2. Stepped-care : description and examples
		3. Lessons from the literature
5	**Clinical decision support - Clinical practice guidelines [CCM: DS]:** Participants will have a common understanding of clinical practice guidelines, as well as their benefits and limitations, and know how to refer to them in their clinical decision making.	1. Clinical decision support
		2. Clinical practice guidelines
		3. Benefits and limitations related to clinical practice guidelines
		4. Where to find clinical practice guidelines
6	**Working together: how to implement strategies for interprofessional collaboration [CCM: DSD, DS]:** To enable settings to identify strategies to offer complementary services, ensure coordination of care, foster teamwork and define modalities that encourage mutual support between providers.	1. Interprofessional collaboration
		2. A continuum of collaborative practices
		3. What is collaborative care in mental health?
		4. Simple changes for depression in primary care
		5. Collaborative care models for anxiety and depressive disorders
		6. Case management - a key element of collaborative care
7	**Productive interactions between the patient and health care team[CCM: SM]:** Identify the tools and strategies that best enable care providers to offer accurate information to patients about their health including motivational interviewing and shared decision-making.	1. The role of productive interactions in the Chronic Care Model
		2. Patient education
		3. Motivational interviewing
		4. Shared decision-making: a key to productive interactions
		5. Communication strategies: The communication cycle
8	**Self-management support [CCM: SM]:** To promote the various self-management tools and identify strategies which encourage the implementation of interventions aiming to support self-care among people with depression and/or anxiety disorders.	1. Self-management support: what and how?
		2. The effectiveness of self-management strategies for the treatment of depression and anxiety disorders
		3. The characteristics of a good self-manager
		4. What helps patients succeed in the management of their care
		5. The right form of self-management support for the right patient
		6. Two self-management program examples:
		• Self-management depression workshop
		• Self-care depression guide
9	**Low-intensity interventions and the various group interventions[CCM: DS, SM]: **To provide examples of promising practices and help participants to identify low-intensity interventions that could be offered to people with depression and/or anxiety disorders. To differentiate the various forms of group interventions that can be offered to patients such as group therapy, psychoeducational groups, and self-help and support groups.	1.Low-intensity interventions
		2. Examples of low-intensity interventions
		The different group interventions
10	**Clinical Information Systems [CCM: CIS]:** To demonstrate the importance of clinical information systems in patient monitoring.	1. What is a clinical information system?
		2. The functions of a clinical information system
		3. The indicators to include in the clinical information system
		4. The patient follow-up worksheet

*Abbreviations refer to the Chronic Care Model components implemented in the knowledge application program: HSO = Health System Organization; DSD = Delivery System Design; DS = Decision Support; CIS = Clinical Information Systems; SM = Self-Management Support; CRP = Community Resources and Policies.

#### The clinical information systems strategy

The collection of clinical data on patients with mental health problems is considered essential to achieve success in quality improvement efforts from a population-based approach [66,67]. Establishing a basic clinical information system for patients with anxiety and depressive disorders is mandatory to the participation of each site in the research project, as patient data on processes of care and health outcomes help plan and coordinate interdisciplinary patient care and obtain feedback for specific population groups [67]. Due to the considerable time, efforts and resources required to implement a clinical information system, as well as contextual risk factors, the strategy preconized in this project is to actively support CMHTs in the implementation of a basic data collection approach [68]. A patient data collection procedure will be introduced at each CMHT to support the follow-up, practice evaluation, and quality of care for patients with anxiety or depressive disorders.

The training module will present a patient data collection tool as well as a procedure for the implementation of a basic clinical information system in each CMHT. Medical clinics will be provided with the same toolkit if clinicians are also interested in collecting patient data as part of their collaboration with the CMHTs. The data collection tool will be developed by the research team to gather information on processes of care and health outcomes for patients with anxiety and depressive disorders. We will recommend that health care providers use the data collection tool for their patients aged 18 years or older with a new episode of major depression, panic disorder with or without agoraphobia, social phobia, and/or general anxiety disorder according to the DSM-IV-TR (primary diagnosis) [69]. These common mental disorders have been selected due to their high prevalence in primary care, their adequate response to both psychological and pharmacological treatments [20,70,71], and because their symptomatology can be monitored with common standardized tools [72,73]. Data on the care process will include the professional consulted, diagnosis, intervention type, referral to a specialist, and patient education. Data on patient health status will include standardized symptom and functioning scales to be filled out by patients on a regular basis. The Patient Health Questionnaire (PHQ-9) [73] for major depression and the Generalised Anxiety Disorder–7 item scale (GAD-7) [72] will be recommended to monitor symptomatology due to their satisfactory psychometric properties, brevity and free access for clinicians. The PHQ-9 is a well-known measure used to evaluate the severity of depression in clinical practice, and the GAD-7 fulfils the same purpose for anxiety disorders. Patients will also complete the Sheehan Disability Scale to evaluate their functioning in three aspects of daily life (work, social life and recreation, family life); this scale has high internal consistency and sensitivity [74,75].

The research team will also provide a series of quality indicators regarding evidence-based practice for anxiety and depression as presented in the knowledge application program on the following topics: assessment; patient education; self-management support; low intensity interventions; pharmacological management; psychotherapy; systematic follow up; and collaborative care. A primary goal is to encourage the establishment of a local quality monitoring routine closely related to their improvement efforts for anxiety and depression. For instance, a quality indicator could be the ‘percentage of patients with depression whose symptoms are reassessed with the PHQ-9 within three months of initiating treatment’ [76], and the local improvement target could be determined by the local working groups. Consistent with the overall approach of our knowledge application approach, the local working groups will have the opportunity to adapt the recommended strategy to their context in terms of patient selection, data collection tool, procedures, quality indicators, and monitoring.

#### The facilitation approach

Evidence encompasses various sources of knowledge, the assumption of which is reflected in the program by the presentation of a knowledge application program based on research evidence, as well as a facilitation process that allows for developing a shared vision of evidence with consideration of clinical experience and local context of each CMHT working group. Facilitation is the key strategy to guide the local CMHTs in the implementation of evidence-based practice for anxiety and depression in clinical practice, and several strategies are introduced throughout the knowledge application program to ensure the success of implementation of evidence in clinical practice. A knowledge broker (HB) with a helping and enabling role, as suggested by the PARiHS framework [56,57], assumes the primary responsibility of external facilitation with each of the local leaders and working groups. The role of the knowledge broker includes: the preparation of the knowledge application program, the presentation of the training modules to the local working groups, and the support of local working groups throughout the project’s planning and implementation stages. The following attributes were considered essential to the role of knowledge broker in the project: communication skills, problem solving skills, and understanding of the mental health service organization in the provincial primary care context. The knowledge broker is actively supported in her role by a team of researchers and collaborators, who help develop and implement the knowledge application program and attend particular meetings as content experts.

The internal facilitation is under the responsibility of a local leader working with a committee. The local leader is preferably a health care manager in the CMHT with project management skills and decision-making authority at the local level. The local working group is composed of seven to fourteen members with the decision-making authority or influence necessary to support the development and implementation of the local quality improvement plan (Table 1). Throughout the knowledge application program, group members work towards building a local quality improvement plan (continuously planned over the course of the local meetings). Centralized regular meetings with the local leaders and the research team complement the facilitation process and provide an opportunity to discuss local implementation strategies among group leaders and researchers.

#### The local quality improvement plans

Although the knowledge application program is structured, it is not a prescriptive approach with regard to the implementation of evidence. The program includes several strategies to improve the quality of primary mental health care and services for anxiety or depressive disorders. These strategies are not mutually exclusive; they are complementary. The program promotes an approach that is tailored to the specific context of each CMHT and collaborating primary care medical clinic. It translates into the various players taking an active role at each project phase. The local quality improvement plans are therefore tailored according to the interaction of evidence, context and facilitation in each CMHT, and local working groups have the responsibility to take into consideration these elements in order to decide which specific improvement strategies they will seek to implement in their local context. The minimal requirement for the local working groups is to include at least two improvement strategies in their local quality improvement plans, as well as the mandatory clinical information systems strategy. This quality improvement plan must include the intervention strategies selected, reasons behind these choices, resources and tools required, approaches to take, a realistic implementation timetable and budget, task distribution, and indicators to assess the success of intervention implementations.

### Phase 2: The implementation of the local quality improvement plans

Following the knowledge transfer phase and preparation of local quality improvement plans, the six local working groups in the CMHTs begin the implementation of their local improvement strategies. The role of the knowledge broker during the implementation phase includes quarterly meetings with local working groups to promote clinical and organizational quality of care assessment, tools and material presented to the community to aid the process, and support to implement Plan-Do-Study-Act cycles [77,78] to encourage experimentation with small changes, gradually progressing towards more complex improvements in each of the exposed organizations throughout the two years of implementation. The knowledge broker will facilitate discussion meetings with local working groups on the local quality improvement targets based on patients’ data collected by each CMHT. A research assistant will be available to provide support, if required by the local working committees, with the aggregation of data and descriptive analysis for the CMHTs prior to each local quarterly meeting. Data will be de-identified before being transmitted to the research team.

### Planning the study of the intervention

#### Data sources

Data collection and analysis will be performed using concurrent mixed methods [79,80] that rely on multiple data sources to facilitate understanding of a complex phenomenon. The mixed data collection method is based on four main data sources. First, a daily log will be kept by a knowledge broker for the whole duration of the research project, which will include meeting reports with local working groups and other contacts with local leaders, summaries of telephone conversations, follow-up discussions with key actors, and field notes. Second, the written documents regarding the local quality improvement plans will be consigned for the analysis with all other relevant case documents. Third, the Assessment of Chronic Illness Care (ACIC) [81] will be used to document the level and nature of changes regarding the six components of the Chronic Care Model. The ACIC is a quality-improvement tool for improving care at the community, organisation, practice and patient levels. The scale is composed of 28 items assessing levels of implementation of quality improvement strategies divided into six sections that are related to the six elements of the Chronic Care Model. The ACIC has been shown to be responsive to organisational quality improvement efforts [81]. The content of the ACIC tool has been adapted by the research team to identify areas of improvement relating specifically to evidence-based care for common mental disorders. The ACIC questionnaire will be completed before and after the implementation of the quality improvement program in each CMHT by a health care manager and a clinician. Fourth, the patient data collection in each CMHT will gather information on processes of care and health outcomes for patients with anxiety and depressive disorders for each case, and will provide information on local quality indicators and improvement targets.

#### Data coding and analysis

The first objective of the study aims at evaluating the implementation of the knowledge application program for anxiety and depressive disorders in the six CMHTs. The second objective aims at examining the contextual factors associated with the selection of quality improvement strategies integrated in the local quality improvement plans, while the third objective aims at exploring barriers and facilitators associated with the level of implementation of the local quality improvement plans. Data coding and reduction for these objectives will be based on all relevant qualitative and quantitative data for each of the six sites. The data management and reduction for all written material will be carried out using NVivo 10 (QSR International) qualitative data analysis software. The coding strategy will be based on the three elements of the PARiHS conceptual framework (evidence, context, facilitation), as well as the variables associated with each of the six components of the Chronic Care Model, and emerging themes over the course of the project. Data will be sorted with the ‘case’ as main analysis unit to allow an in-depth analysis of each case and to compare cases within the continuous analysis process.

The qualitative analysis of the data will be conducted using Miles and Huberman’s iterative cyclical process [80]. Data triangulation involving the first three data sources and the different users will ensure the validity of the description on the cases and their evolution, as well as permit the integration of both data types in the thematic analyses, for a better understanding of the implementation and results in each context. A cross-case analysis will then be conducted toward the end of the study to discern the common themes across cases and examine the interaction of evidence, context and facilitation as described in the PARiHS framework. The systematic comparative analysis of the six study cases will allow for discriminating factors associated with the implementation’s success, and examining the degree of implementation of the knowledge application program components in various contexts. We need to identify which specific components of the program were chosen and which intervention strategies were selected in the local quality improvement plans, whether they were successfully implemented by the end of the implementation phase and what local working groups think about the usefulness of this program and of its different components. We will also examine the contextual factors that influenced the selection of quality improvement strategies in relation to each of the CCM components, as well as explore barriers and facilitators associated with the level of implementation of specific anxiety and depressive disorder management strategies for each of the CCM components as planned in the local quality improvement plans.

The fourth objective aims at assessing the impact of the Clinical Information Systems component of the knowledge application program on the development and implementation of local quality monitoring strategies. Data coding and analysis will be conducted in continuity with the procedure exposed for the previous objectives, with the addition of the fourth data source: data from local clinical information systems. We will examine whether cases successfully implemented a basic clinical information system and introduced a monitoring approach to assess the quality of care for anxiety and depression in their local contexts. We will also explore the use of quality indicators and improvement targets to monitor the implementation of quality improvement strategies as described in the local plans. Contextual factors associated with data collection, selection and adaptation of recommended quality indicators will also be examined, and barriers and enablers to the implementation of the basic clinical information system will also be addressed. The analysis will draw on all data sources, particularly the quality monitoring and quarterly facilitation meetings during the implementation phase in each CMHT. Data for each case will be used for a comprehensive examination of elements, including the coverage of the data collection at the local level, the patient profiles, as well as the types of care processes and patient outcomes data collected. Because the multiple case study aims to provide an in-depth assessment of the implementation process in each specific context, the achievement of quality monitoring goals will be integrated to each within-case analyses.

#### Time frame of the study

The study will be conducted from November 2011 to October 2014. The project’s preparation phase (November 2011 to August 2012) involves preparing the knowledge application program, organising an initial centralized meeting with local leaders and planning local working groups.

During the knowledge transfer phase (September 2012 to January 2013), the ten modules of the knowledge application program are presented to each of the six local working groups over the course of six three-hour meeting and training sessions. Each site then prepares their local quality improvement plan, which establishes their quality improvement strategies. In this plan, they must determine a project timeline, identify and prioritize the required means and resources to support the implementation of their selected strategies, appoint collaborators and prepare a budget. Between February 2013 and March 2013, the second centralized meeting with local leaders will be held in order to assist them in the finalization of their respective local quality improvement plan.

During the implementation phase (April 2013 to October 2014), the research team will provide tools and support as needed to the local working groups in the implementation of their local improvement strategies. PDSA cycles will be introduced, and experimentation with the patient follow-up worksheet will begin. Local meetings will be held at three-month intervals in order to provide an update, collect patient data, and offer feedback about program impacts. A centralized meeting with local working groups will be held at the beginning and at the end of the implementation phase. Data will be collected throughout the study phases. Results will be presented in conferences and scientific journals, as well as to an advisory board involved in an integrated knowledge transfer approach throughout the process (September 2013 to April 2014).

## Discussion

The implementation of an evidence-based knowledge application program for anxiety and depression in primary care aims to improve the organisation and delivery of mental health services from a population perspective. This project will provide in-depth understanding regarding the contextual factors that are associated with the selection of strategies for improvement, the barriers and facilitators throughout the implementation process, and the development of a quality monitoring routine at the local level. The project will allow a close examination of the interplay between evidence, context and facilitation as conceptualized in the PARiHS framework. The project’s integrated approach to knowledge application is at the core of this collaborative endeavour, one that involves clinicians, health care managers, researchers, and decision-makers alike. This approach allows all stakeholders involved to develop a shared perspective on mental health, which is a determining factor in regard to the impact of the program on primary mental health care practices in Quebec [82]. We believe this collaboration to be conducive to enhancing reflective practices focused on the quality of mental health care.

A set of limitations should be considered for this study protocol. First, the research design was conceived primarily to examine conceptual and instrumental knowledge use rather than to examine the effects of multifaceted interventions adopted and implemented by each case following the knowledge transfer phase [83]. Therefore, it will not be possible to draw conclusions on changes in patient outcomes from the implementation of the knowledge application program. However, the results of the multiple case study will provide critical information to guide the development of a structured tailored organisational and professional intervention that could be examined with a pragmatic randomized controlled trial in other CMHTs and medical clinics to evaluate the effects of the implementation of evidence-based practice. Second, since the study is conducted in CMHTs and medical clinics in the province of Quebec, Canada, caution will be required in the transferability of findings to other primary care settings or organizations. We attempted to maximize the generalizability of results for our provincial health care system by conducting the multiple case study in six different contexts and primary care settings. Third, the conduct of an implementation study in multiple routine clinical settings presents challenges for data collection and consequently may present a threat to validity, which we addressed by using a triangulation approach based on multiple quantitative and qualitative data sources.

The uptake of evidence to improve the quality of care for common mental disorders in primary care is a complex process that requires careful consideration of the context in which innovations are introduced. The implementation of the knowledge application program within a population health perspective is consistent with the priorities set forth in the current mental health care reform in Quebec. Strengthening primary mental health care will lead to a more efficient health care system.

## Competing interests

The authors declare that they have no competing interests.

## Authors’ contributions

All authors read and approved the final manuscript.
